# Large chromosomal deletions and impaired homologous recombination repairing in HEK293T cells exposed to polychlorinated biphenyl 153

**DOI:** 10.7717/peerj.11816

**Published:** 2021-07-28

**Authors:** Jiaci Li, Yaqing Jing, Yi Liu, Yawei Ru, Mingyan Ju, Yuxia Zhao, Guang Li

**Affiliations:** Tianjin Medical University, Tianjin, China

**Keywords:** PCB153, HEK293T cells, Copy number variation, Homologous recombination repair, DNA damage

## Abstract

**Background:**

Polychlorinated biphenyls (PCBs) are persistent pollutants with carcinogenesis and mutagenesis effects which have been closely associated with PCBs-induced DNA damage. However, the detailed DNA damage events and corresponding pathway alterations under PCBs poisoning is still not well understood.

**Methods:**

Whole-genome sequencing (WGS) and RNA sequencing (RNA-seq) were used to explore genome wide variations and related pathway changes in HEK293T cells that challenged by 15 µM PCB153 for 96 h *in vitro*. Double strand breaks (DSBs) were measured by 53BP1 foci detection, altered pathways were confirmed by quantitative real-time PCR (qPCR).

**Results:**

The results indicated that abundant copy number variations (CNVs), including four duplications and 30 deletions, occurred in PCB153-exposed HEK293T cells. Multiple large fragment deletions (>1 Mb) involving up to 245 Mb regions on many chromosomes. Missense mutations were found in six tumor susceptibility genes, two of which are key members participating in homologous recombination (HR) repair response, *BRCA1* and *BRCA2*. RNA-seq data showed that PCB153 poisoning apparently suppressedHR repairing genes. Besides, 15 µM PCB153 exposure significantly increased 53BP1 foci formation and effectively reduced *BRCA1*, *RAD51B* and *RAD51C* expression, indicating an elevated DSBs and impaired HR repairing.

**Conclusion:**

This study firstly reported multiple large chromosomal deletions and impaired HR repairing in PCB153-exposed HEK293T cells, which provided a new insight into the understanding of early response and the mechanism underlying PCB153 genotoxicity. The chromosomal instabilities might be related to the impaired HR repairing that induced by PCB153; however, further investigations, especially on actual toxic effects of human body, are needed to confirm such speculation.

## Introduction

As widespread persistent organic pollutants, Polychlorinated biphenyls (PCBs) had been widely used in industrial manufacturing in the past few decades because of their high stability, heat resistance, and high dielectric constant. Although the commercially production and usage were banned in 1970s–1980s, there is still a high environmental load since these persistent chemicals are stable in the environment and can be accumulated in plants and animals. In the past several decades, health problems, including endocrine disorders, neurotoxicity, gonadal toxicity, and carcinogenicity of PCBs exposed populations, have made PCBs exposure a public concern (Lauby-Secretan et al., 2013).

Based on the chemical structure, PCBs are classified into non-ortho-substituted and ortho-substituted congeners (Dervola et al., 2015). The toxic effects of PCBs people and congeners have been extensively explored (Dervola et al., 2015). Non-ortho-substituted PCBs are structurally similar to dioxin and their toxicity and mode of action are well-established (Aluru et al., 2020). In contrast, very little is known about the effects of ortho-substituted PCBs (Aluru et al., 2020). Recent investigations have suggested that DNA damage caused by PCBs poisoning is largely responsible for their adverse effects especially carcinogenesis (McAuliffe et al., 2012). Dong et al. (2014) reported that PCB-quinone, one of PCBs metabolites, which exposure triggered obvious DNA strand breaks and chromosome breaks in HepG2 cells. Our previous report showed increased DNA adducts, chromosome aberrations, dominant micronucleus formations, and downregulations of some DNA homologous recombination (HR) repairing pathway genes in PCBs exposed males (Wang et al., 2018). These works suggested DNA single and double strand breaks post PCBs poisoning, however, most of the findings were descriptions of genotoxic outcomes after PCBs exposure rather than specific DNA damaging events. Detailed information on DNA damaging is needed to revealing the genetic toxicological mechanism of PCBs.

The objective of this study is to investigate the effects of PCB153 (2,2′,4,4′,5,5′-hexachlorobiphenyl) exposure on HEK293T cells. PCB153 is a widely distributed ortho-substituted PCB with great stability and tendency to bioaccumulate in lipid-rich tissues (Urbani et al., 2021; Aluru et al., 2020). It is currently considered as one of the most persistent PCBs and the biological effects of PCB153 have attracted much attention (Urbani et al., 2021; Aluru et al., 2020). Although several studies have highlighted its neurotoxicity and metabolic toxicity, much more investigations are still needed to understand the genotoxic effects and mechanisms of PCB153 exposure (Enayah et al., 2018; Coccini et al., 2011). In the present study, the detailed DNA damage events and corresponding pathway alterations of HEK293T cells were identified using whole-genome sequencing (WGS) and RNA sequencing (RNA-seq), after 15 µM PCB153 treatments for 96 h **in vitro*.* The genome variations and related pathway changes could provide new evidences for understanding the genotoxicity of PCBs.

## Materials and Methods

### Materials

**Figure 1:**
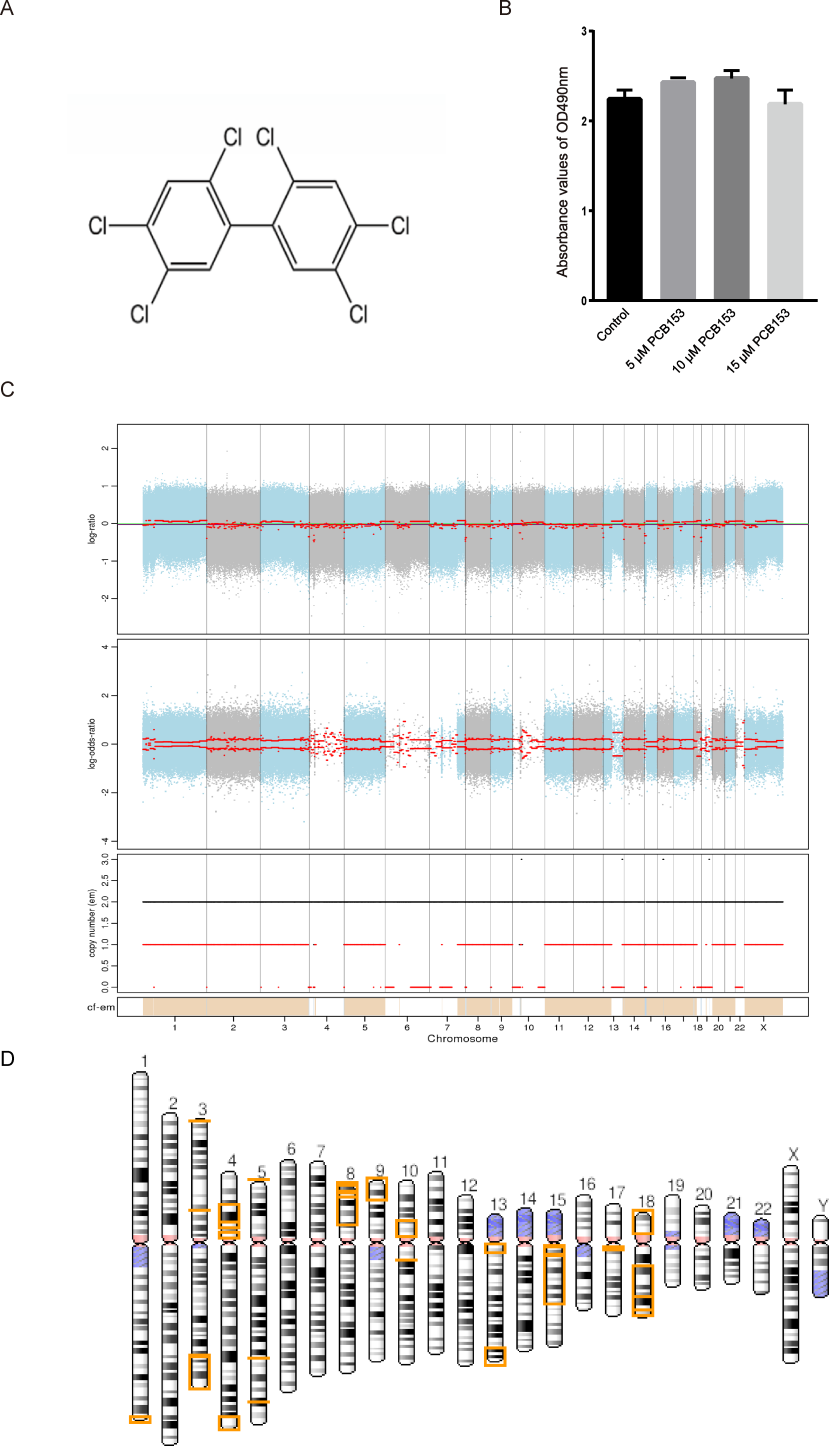
Copy number variation (CNV) analysis in PCB153-exposed HEK293T cells. (A) Chemical structure of 2,2′,4,4′,5,5′-hexachlorobiphenyl (PCB153). (B) The proliferation of HEK293T cells measured using MTT 48 h post different dosage of PCB153 treatment, and 15 µM PCB153 did not affect the cell proliferation. *n* = 6 for each group. (C) Copy number variation (CNV) analysis in PCB153-exposed HEK293T cells determined by whole-genome sequencing (WGS). The Top and middle pictures are the log2 value of reads coverage depth ratio of two samples at different positions of chromosomes. The red line is the result after correction, and the value of 0 represents the absence of CNV. In the bottom picture, when the value of the black line is greater than 2, the copy number increases; when the value of the red line is less than 2, the copy number decreases. (D) The genome-wide CNV cases were abundantly found in PCB153-exposed HEK293T cells. There were multiple large fragment deletions on many chromosomes as shown above. The lost genetic fragment within each CNV region were shown in the yellow color on the chromosomes. For full list of annotated genes, please see the Supplemental Information.

All cell culture medium and components, such as Dulbecco’s modified Eagle’s medium (DMEM) and penicillin-streptomycin (PS) were provided by Invitrogen (Waltham, MA, USA). Fetal Bovine Serum (FBS) was obtained from Thermo Fisher Scientific (Waltham, MA, USA). Dimethyl sulfoxide (DMSO) was purchased from Sigma (St. Louis MO, USA). PCB153 (molecular weight, 361 g/mol; purity, 99.9%) (Fig. 1A) was provided by Accustandard (USA).

### Cell culture

HEK293T cell line provided by Professor Feng Wang (Tianjin Medical University) was used in the present study. The HEK293T cell line was cultured in high-glucose DMEM with 10% FBS and 1% PS in a humidified apparatus at 37 °C with 5% CO_2_ (Lu et al., 2016).

### HEK293T cells exposure in PCB153

A stock solution of PCB153 was dissolved in DMSO and 0.05% DMSO is the final concentration after adding into cell culture medium. HEK293T cells were treated with PCB153 at different concentrations of 5 µM, 10 µM, and 15 µM (Eum et al., 2009; Xin et al., 2016). Control cultures only received the solvent. Cells were collected after 48 h and 96 h of culture. For genome and RNA sequencing, HEK293T cells exposed to 15 µM PCB153 for 96 h were used.

### Cell viability examination

The cell viability was measured using 3-(4,5-dimethylthiazol-2-yl)-2,5-diphenyltetrazolium bromide (MTT) test. The HEK293T cells were seeded in 96-well plates at 1  × 10^4^ cells per well. The gradient concentrations of PCB153 were added after 24 h. The HEK293T cells treated with PCB153 were continually cultured for 24 h. Totally 20 µL of MTT solution (0.5% MTT) were added and incubated for 4 h. 150 µL of DMSO was added to each well to dissolve formazan crystals. The light absorption value was measured at 490 nm.

### Whole-genome sequencing (WGS)

293T cell precipitations were randomly interrupted by the High Performance Ultrasonic Sample Processing System (COVARIS), and fragments of about 350bp were obtained after fragment selection. The end of the DNA fragment was then repaired by adding “A” base to the 3 ’end and adding library connectors to both ends. Linear amplification (LM-PCR) was performed on the ligated libraries. After rolling circle amplification (RCA), the DNA Nano Ball (DNB) was generated from the library. After qualified quality control, the DNA Nano Ball (DNB) could be processed by computer. We used the BGISEQ-500 platform to perform high-throughput sequencing on each qualified library and ensure that the data volume of each sample was up to standard.

### Total RNA extraction and RNA sequencing (RNA-seq)

Total RNA was extracted from cell precipitations using Trizol (Invitrogen, Carlsbad, CA, USA) according to manual instruction. Before detection by Fragment Analyzer, RNA samples were thawed on the ice, thoroughly mixed and centrifuged. Standard Sensitivity RNA Analysis Kit (15 nt) (DNF-471) was used to detect the samples, and the samples were diluted 3–8 times at a detection concentration of 400–1,000 ng/ µL. RIN values of all samples were greater than or equal to 7.0 and 28S/18S ratios were greater than or equal to 1.5, indicating that the extracted RNA was of good quality and met the requirements of sequencing. After sequencing data quality control and filtering, we compared the filtered clean reads to the reference sequence. After the alignment, whether the alignment results passed the second quality control (QC of alignment) was determined by statistical analysis of alignment rate and distribution of reads in reference sequences. In the case of secondary quality control, quantitative gene analysis and various analyses based on gene expression level (differential gene screening, etc.) were carried out, and differentially expressed genes were detected among the screened samples.

DNBSEQ platform was used for RNA-sequencing. Pathway enrichments were carried out by the phyper function in R software according to KEGG pathway annotation classifications. Then *Q* value was obtained by FDR correction of the *P* value, and *Q* value ≤ 0.05 was usually considered as significant enrichment. According to Heatmap, Bowtie2 was applied to compare clean reads to the reference gene sequence, and then RSEM was used to calculate the gene expression level of each sample. The DESEQ2 method is based on the principle of negative binomial distribution. The method described by Love et al. was used to conduct differentially expressed gene (DEG), which is a test for a gene whose expression level varies between samples (Love, Huber & Anders, 2014). According to the results of DEG detections, the differential genes were performed by using R-package pheatmap for hierarchical clustering analysis.

### Immunofluorescent staining

Immunofluorescence tests were used to measure 53BP1 expression as described in previous study (Noordermeer et al., 2018). We fixed the cells with 2% paraformaldehyde for 10 min, permeabilized with 0.5% Triton X-100 in PBS for 20 min and incubated with blocking solution (1% bovine albumin V, 1% gelatin in PBS) for 1 h. The cells were incubated with 53BP1 antibodies (1:5000 dilution; Novus Biological, Littleton, CO, USA) for 2 h. After washing in 0.1% Tween 20 in Tris-buffered saline (TBST), the cells were incubated for 30 min with secondary antibodies (1:5,000 dilution; Life Technologies, Carlsbad, CA, USA) in the dark. After dehydrated in ethanol, the cells were mounted with 4′,6-diamidino-2-phenyl-indole (DAPI, D3571; Life Technologies, USA). The positive signals were observed and counted by using fluorescence microscopy (Nikon, Japan).

### Real-time quantitative PCR (qPCR) analysis

The mRNA expression of *BRCA1*, *RAD51B*, and *RAD51C* were examined by qPCR. The total RNA was reverse-transcribed into cDNA with a reverse transcription system (Promega, Madison, WI, USA) consistent with the manufacturer’s protocol. The objective sequences of genes to be observed were amplified with SYBR green master mix (QIAGEN, Germany) using the Rotor gene Q 2000 (QIAGEN, Germany). The primers were listed in Table S1. The level of *GAPDH* served as control for the data analysis.

### Statistical analysis

FACET software was used to detect Somatic CNV in Tumor and Normal pairs. Phyper function in R software was used for enrichment analysis to calculate *p* value, and then *Q* value was obtained by FDR correction of *P* value, *Q* value ≤ 0.05 was considered as significant enrichment. Bowtie2 was used to compare clean reads to the reference gene sequences, and then RSEM was used to calculate the gene expression level of each sample. The DESEQ2 method was used to conduct DEG for differentially expressed genes. The union of differential genes was performed by using R-package pheatmap for hierarchical clustering analysis.

All experiments were repeated at least three times. The results were expressed as mean ± standard deviation (SD). The student’s *t*-test was used for comparisons between two groups, and one-way ANOVA was used for comparisons among multiple groups by IBM SPSS Statistics 21. Differences with *P* < 0.05 was considered statistically significant.

## Results

### PCB153 poisoning caused multiple large fragment deletions on many chromosomes

The cytotoxicity of PCB153 on HEK293T cells was analyzed by MTT. Compared with 0.05% DMSO-treated cells, PCB153 did not affect cell viability (Fig. 1B). To explore the genotoxic events caused by PCB153, copy number variations (CNVs), single nucleotide variants (SNVs), and insertions and deletions (Indels) were determined by WGS. The results indicated that PCB153 poisoning generated apparent change of CNVs, SNVs and Indels in the whole-genome (Tables S2–S6).

There were 34 large (>1 Mb) copy number variations (CNVs), including 4 duplications (282.6 Mb) and 30 deletions (245.4 Mb) were identified in PCB153-exposed cells (Fig. 1C, Tables S2–S4). It was shocking that CNVs covered multiple chromosomes and large fragment deletions could be observed on many autosomes in PCB153-exposed HEK293T cells (Fig. 1D). And more than half of the fragment deletion events were homozygote deletions (Table S4). For example, terminal deletions from the short arm of chromosome 9 and chromosome 18 generated about 18 Mb and 15 Mb length fragments loss, respectively, with lots of genes affected (Figs. S2, S3).

### PCB153 poisoning induced missense mutations of 6 tumor suppressor genes

Germline mutation information was also analyzed through comparing the detected mutant genes with the CGC (Cancer Gene Censue) database using GATK software. And there were missense mutations located in 6 tumor susceptibility genes, including *PTCH1*, *BRCA2*, *BLM*, *ERCC4*, *BRCA1,* and *SETBP1* (Table S7), whose function loss have been closely associated with several hereditary tumor susceptibility syndromes (Acuna-Hidalgo et al., 2017; Qian et al., 2017; Romagnolo, Romagnolo & Selmin, 2015; Zhang et al., 2018). Interestingly, *BRCA1* and *BRCA2* are key members participating in homologous recombination (HR) repair upon DNA damage and the missense mutations were likely to affect the HR repair potential.

### PCB153 exposure inhibited HR repair in HEK293T cells

**Figure 2:**
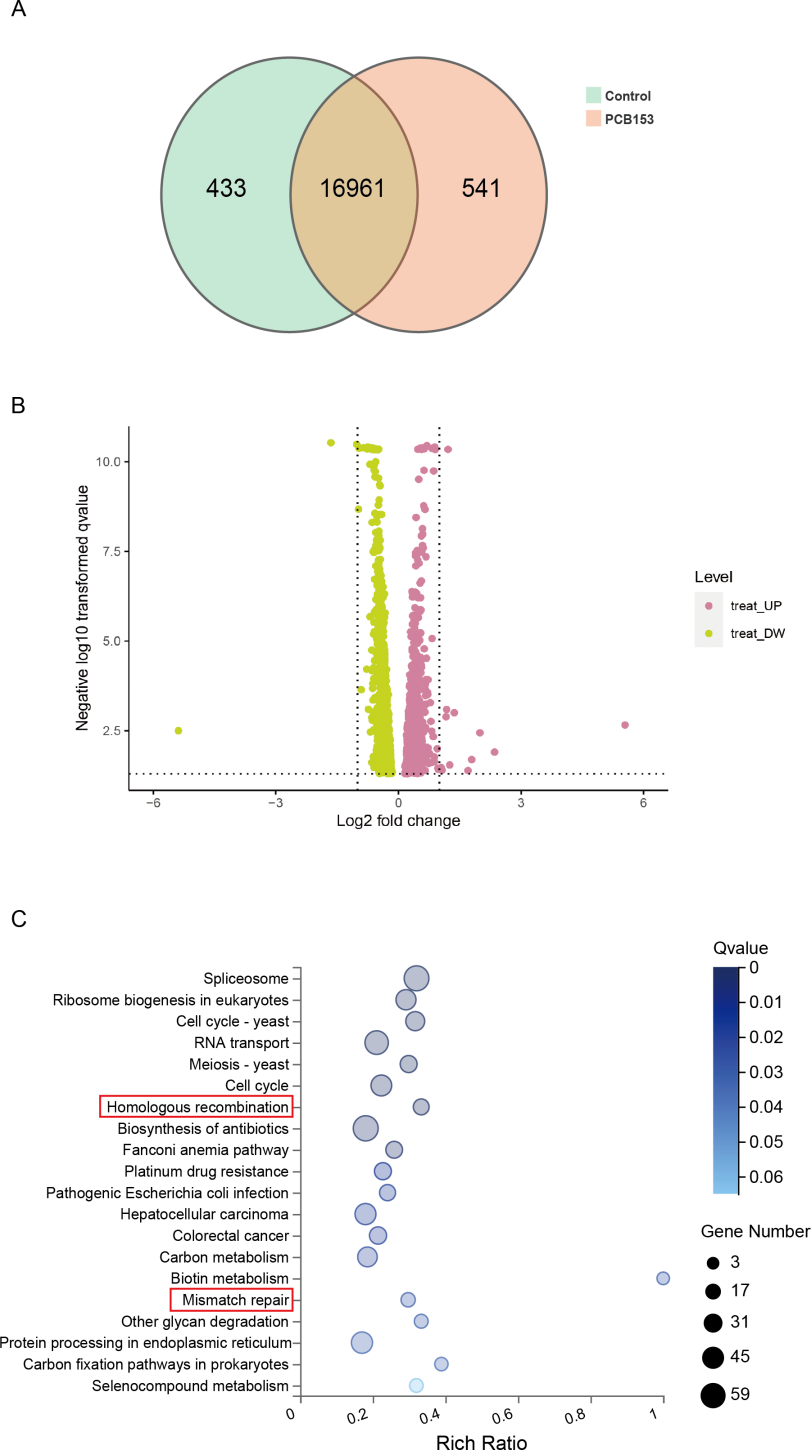
KEGG pathway enrich analysis in PCB153-exposed HEK293T cells. (A) The number of differentially expressed genes between PCB153-treated cells and control group showed by VENN diagram. (B) Up-regulated and down-regulated genes between PCB153-exposed cells and control group displayed by Volcano plot. The pink circles represented up-regulated genes, and the yellow circles represented down-regulated genes. (C) KEGG pathway analysis of the differentially expressed genes in PCB153-treated HEK293T cells. The number of genes were marked as bubble sizes. Colors represented enrichment Q value, and darker colors represented smaller Q value.

**Figure 3:**
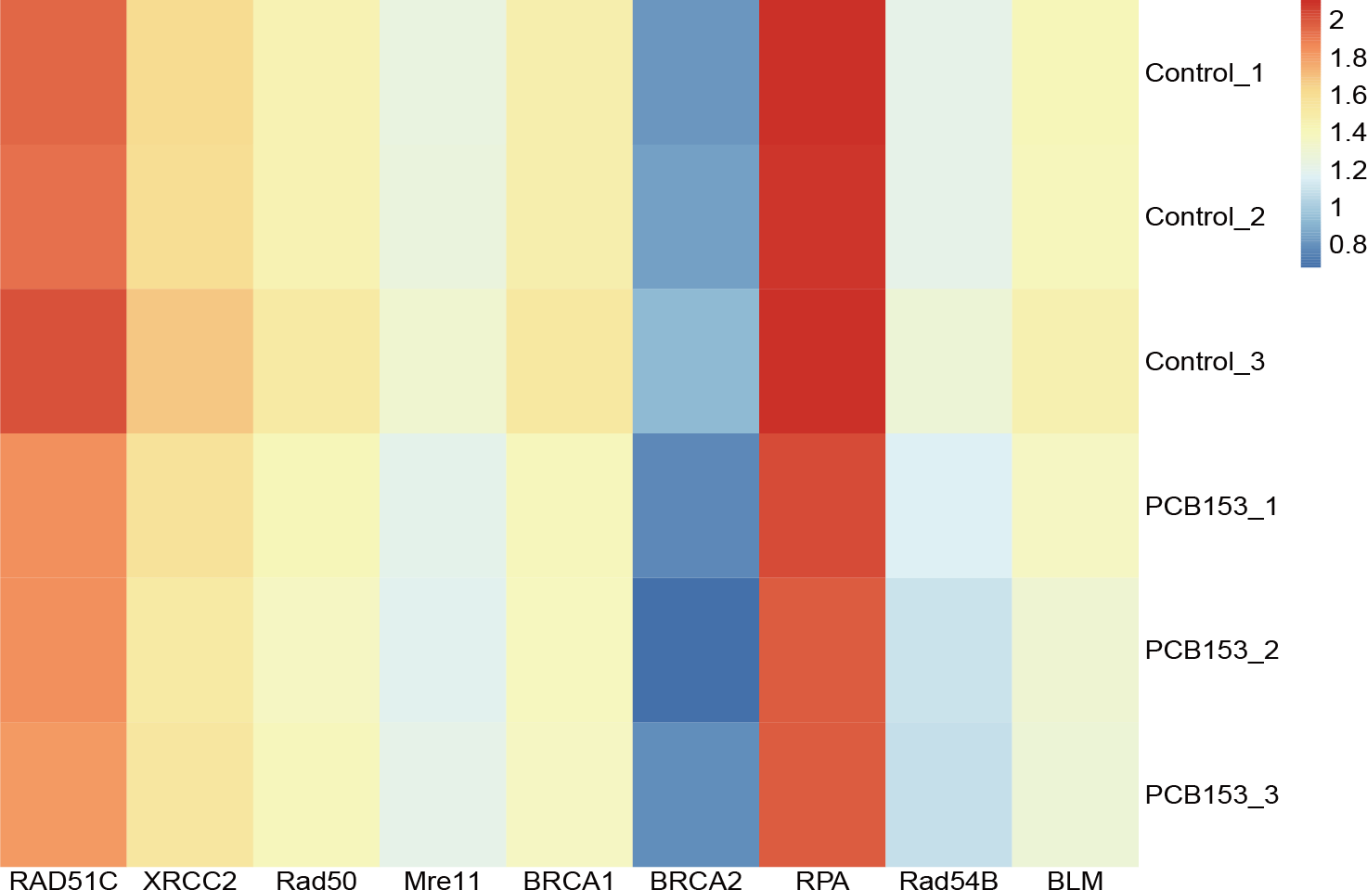
PCB153 inhibition of homologous recombination repair in HEK293T cells. The heatmap showed the expression of genes participating in homologous recombination repair.

RNA-seq was applied to examined the altered pathways that might be related to the genotoxicity of PCB153 exposure. Figure 2A showed a VENN diagram of the overlapping genes between PCB153 exposed cells and control samples. The sequencing data showed 1051 up-regulated and 1622 down-regulated DEGs (volcano plot, Fig. 2B). The enriched pathways included ‘homologous recombination repair’ (KEGG Term ID 03440) and ‘mismatch repair’ (KEGG Term ID 03430) (volcano plot, Fig. 2C). And most of the genes involved in the two cascades were downregulated (Fig. 3, Fig. S1), suggesting the inhibited HR repair and mismatch repair responses. The reduction of DNA damage repair ability will probably lead to severe mutations.

### PCB153 poisoning caused DNA double-strand break (DSB) and HR repair genes downregulation in the HEK293T cells

**Figure 4:**
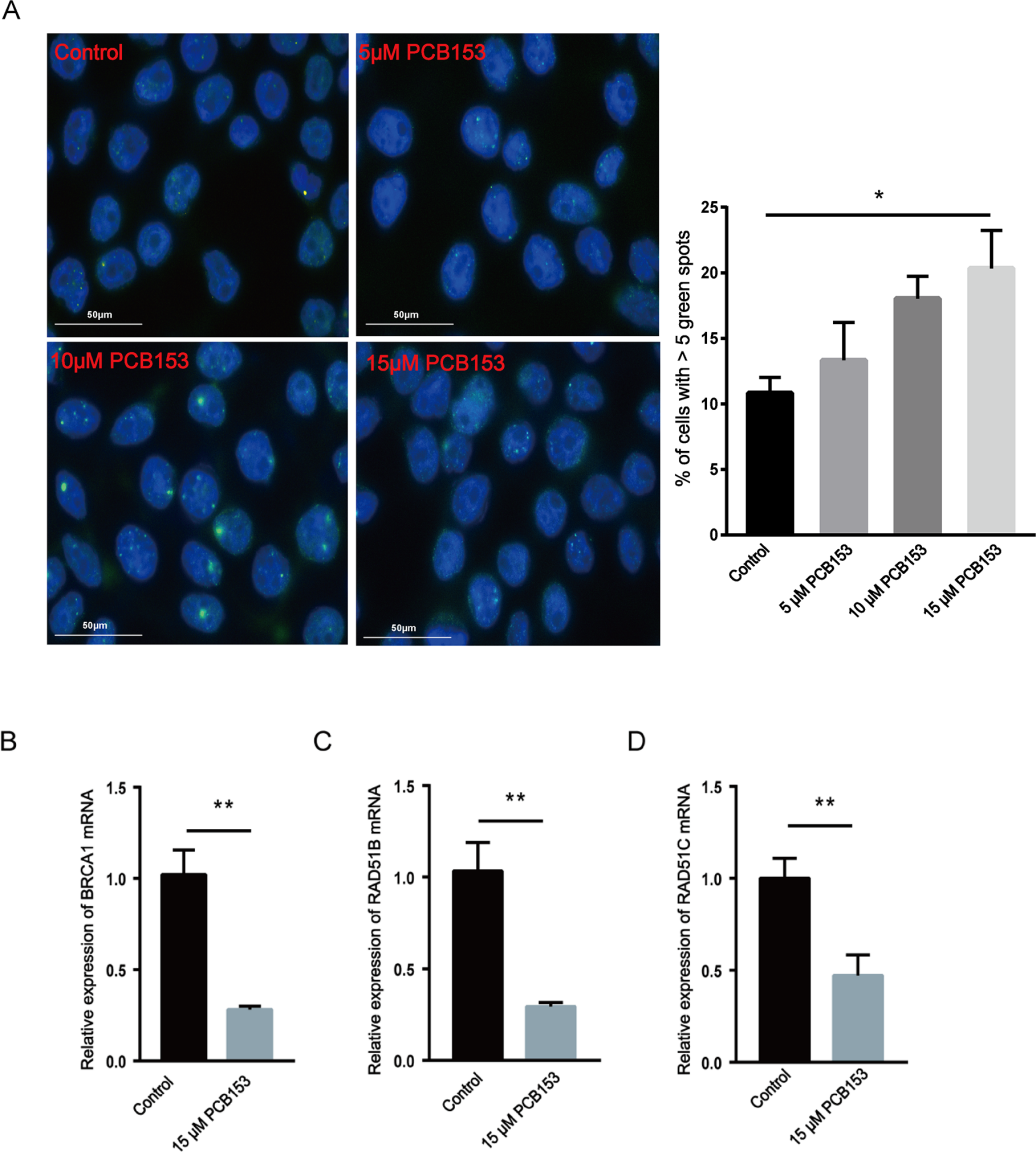
Assessment of DNA damage and HR repair in PCB153-exposed HEK293T cells. (A) The expression of 53BP1 in HEK293T cells after PCB153 exposure measured using immunofluorescence. Blue fluorescence is DAPI and green is 53BP1. (B–D) Relative mRNA expressions of BRCA1, RAD51B and RAD51C under 15 µM PCB153 treatment assessed using qPCR. The control cells were treated with 0.05% DMSO. Mean ± SD was shown (*n* = 3). Similar results were observed in at least three independent experiments. * *p* < 0.05, ** *p* < 0.01.

The content of 53BP1, a marker of DSBs, was examined to show the effects of PCB153 poisoning on DNA damage in HEK293T cells **in vitro**. The 53BP1 foci assay showed observably increased 53BP1 signals after PCB153 exposure compared to the control cells (Fig. 4A), indicating the aggravated DSBs. The mRNA expression of some important HR repair genes were determined by qPCR. Consistent with the RNA-seq results, the expression levels of *BRCA1*, *RAD51B,* and *RAD51C* that involved in HR repairing were all significantly decreased after 15 µM PCB153 exposure (Figs. 4B–4D). There were no obvious changes in the transcription level of *LIG4*, *XRCC5,* and *XRCC6,* which are responsible for non-homologous end-joining (NHEJ) (Fig. S4). These results demonstrated that PCB153 poisoning caused obvious DNA damage and apparently inhibited DNA HR repair.

## Discussion

PCBs are a group of persistent organic pollutants (POPs) and have been defined as human carcinogens. Studies have demonstrated that the bioaccumulation of PCBs is closely linked to human diseases including cancers (Huang et al., 2019) and early developmental metabolic disorder (Yuan et al., 2018). Since the occurrence of human tumors and other diseases is often accompanied by genetic mutation, the mutagenicity is important in risk assessment and pathogenesis understanding of PCBs. Recently, increasing evidences have showed that PCBs challenge can induce DNA damage and affect DNA damage repair systems, which have been closely associated with tumorgenesis (Dogan & Alcigir, 2019). But most of the reports focused on describing the genotoxic outcomes based on combined exposure and the DNA damage events responding to the pollutants exposure have not been fully explored.

Here, PCB153 was chosen as a representative of the PCBs to treat HEK293T cells since it is currently considered as one of the most persistent PCBs (Aluru et al., 2020; Urbani et al., 2021). WGS and RNA-seq were used to investigate the genome-wide variations and altered pathways in 15 µM PCB153 exposed HEK293T cells. The results showed that abundant copy number variations (CNVs) including 4 increase incidents and 30 decrease incidents occurred post PCB153 exposure (Fig. 1C, Tables S2–S4). Surprisingly, the copy number decreases originated from multiple large fragment deletions involving up to 245,442,338 bps affecting many autosomes (Fig. 1D, Tables S2, S4). Such long fragment deletions led to loss of numerous genes (Figs. S2, S3). Besides, lots of newly found SNPs and Indels could also be detected in PCB153 challenged cells (Table S5–S6). These data suggested severe DNA damage and genome instability. Also, the increased 53BP1 foci formation indicated the elevated DSBs (Fig. 4A).

DNA damage is manifested as a permanent change in DNA nucleotide sequence during the process of replication, and it can cause DSB, gene mutations and chromosome rearrangements (Volkova et al., 2020). DSB signaling and repair is crucial to preserve genomic integrity and maintain cellular homeostasis since DSBs repair failure often leads to gross chromosomal rearrangements such as deletions, translocations and amplifications (So & Martin, 2019). NHEJ and HR are the two main pathways for DSBs repair (Kakarougkas & Jeggo, 2014). NHEJ repair is a quick, DNA sequence homology independent, but error-prone repair mechanism (Rassool & Tomkinson, 2010). On the contrary, HR repair provides precise repairing of DSBs. Thus, DSB repair pathway selection can also influence the genome stability and carcinogenesis.

Consistent with the findings in PCBs exposed tumor cell lines and populations (Rattenborg, Gjermandsen & Bonefeld-Jorgensen, 2002; Wang et al., 2018), the downregulation of HR-related genes existed in PCB153 challenged HEK293T cells, indicating the suppressed HR response (Figs. 4B–4D). While NHEJ related gene expressions were relatively normal, suggested that DNA repairing was switched NHEJ in PCB153 exposed cells. Rassool .et al. have reported that PCB29-pQ could activate NHEJ repair, which may cause aberrant repair of DNA damage and increase the potential risk of carcinogenesis and mutagenesis (Rassool & Tomkinson, 2010). The imbalance between HR and NHEJ in PCB153 exposed HEK293T cells might be one important reason for the large chromosomal deletions. Interestingly, a handful HR gene mutations could also be observed in our present study, seeming that HR genes are more susceptible to PCB damage. However, “selective” mutations were not common in HR genes among PCB exposed subjects. There might be much more mutations or other abnormal changes that had not been captured since gross chromosomal deletions occurred in PCB153 exposed cells. And the defect of HR was mainly due to depressed expressions rather than mutations. In general, the PCB induced DNA damage “signature” might be a combined result of PCB induced mutations and the imbalanced HR/NHEJ repairing, and much more investigations are still needed to explore the involved machanism.

Notably, the HEK293T cells are immortalized cells transfected by adenovirus type 5 DNA carrying some chromosome numerical and structural aberrations. And they are often applied as a model for studying the transforming/oncogenic properties of cancer-associated genes, which do not involve in DNA damage repair pathways (Stepanenko & Dmitrenko, 2015). The original genome instability might make them more sensitive to the PCB153 exposure. Still, the data gave us some inspirations in understanding the genotoxic effects of PCBs.

## Conclusions

The current study firstly reported extensive copy number variations (CNVs) mainly due to multiple deletions of long chromosomal fragments and impaired HR repairing in PCB153-exposed HEK293T cells, which provided new evidences on understanding of early responses and the mechanism underlying PCB153 genotoxicity. The CNV incidents might be probably related to the impaired HR repair response, which needed further investigations to confirm. Since HEK293T cells are not normal human diploid cells, the actual toxic effects on the human body need further research.

##  Supplemental Information

10.7717/peerj.11816/supp-1 Supplemental Information 1 Effects of PCB153 on cell proliferation

10.7717/peerj.11816/supp-2 Supplemental Information 2 QPCR

10.7717/peerj.11816/supp-3 Supplemental Information 3 Raw data exported from qPCR for BRCA1 gene and RAD51B gene

10.7717/peerj.11816/supp-4 Supplemental Information 4 Raw data exported from qPCR for RAD51C gene

10.7717/peerj.11816/supp-5 Supplemental Information 5 Copy number variations in HEK293T cell line exposed to PCB153

## Additional Information and Declarations

### Data Availability

The following information was supplied regarding data availability: Raw 53BP1 immunofluorescence data is available at Figshare: Li, Jiaci (2021): Immunofluorescent staining.zip. figshare. Dataset. https://doi.org/10.6084/m9.figshare.14178254.v1. The raw qPCR and cell proliferation data is available in the Supplemental Files. The WGS data is available at NCBI SRA: PRJNA715855. The RNA-seq data is available at NCBI SRA: PRJNA717680.
