# Supplementary material for: Large chromosomal deletions and impaired homologous recombination repairing in HEK293T cells exposed to polychlorinated biphenyl 153
Source: PeerJ. 2021 Jul 28;9:e11816. doi: 10.7717/peerj.11816 (PMC8325425; doi:10.7717/peerj.11816)
Supplement: Supplemental Information 5 [file peerj-09-11816-s005.doc]

**Supplemental Materials**

**Table S1 Primer sequences used in the qPCR experiments**

| Target gene | Primer sequences (from 5´ to 3´) | Product length |
| --- | --- | --- |
| *BRCA1*  *(BRCA1 DNA repair associated)*  *RAD51B*  *(RAD51 paralog B)*  *RAD51C*  *(RAD51 paralog C)*  *LIG4*  *(DNA ligase 4)*  *XRCC5*  *(X-ray repair cross complementing 5)*  *XRCC6*  *(X-ray repair cross complementing 6)*  *GAPDH*  *(glyceraldehyde-3-phosphate dehydrogenase)* | F: CTGAAGACTGCTCAGGGCTATC  R: AGGGTAGCTGTTAGAAGGCTGG  F: CTGGTCTGAGTTATCGAGGTGTC  R: GCTTCGTCCAAAGCAGAAAGGG  F: GTGAAACCCTCCGAGCTTAGCA  R: CCTGCTCAAGAAGTTCCAGTGC  F: CAGCAGAGATCGTACCCAGTGA  R: TGCGAGCTTACCAGATGCCTTC  F: GTTCTAAAGGTCTTTGCAGCAAGA  R: AAAAGCCACGCCGACTTGAGGA  F: GGTTTCAAGCCGTTGGTACTGC  R: CTCCAGACACTTGATGAGCAGAG  F: CAGGAGGCATTGCTGATGAT  R: GAAGGCTGGGGCTCATTT | 155bp  154bp  160bp  152bp  147bp  129bp  138bp |

**Table S2 Somatic CNV statistical results**

| Samples | Gain count | Gain size | Loss count | Loss size |
| --- | --- | --- | --- | --- |
| Control-VS-15μM PCB153 | 4 | 2826244 | 30 | 245442338 |

Gain count: the number of increased gene copies; Gain size: the size of increased gene copies; Loss count: the number of decreased gene copies; Loss size: the size of decreased gene copies.

**Table S3 Somatic CNV gain results**

| Chromosome | Start | End | Tcn.em |
| --- | --- | --- | --- |
| 10  13  16  19 | 46952900  94826400  29803300  43525500 | 47560619  95498400  31059325  43816000 | 3  3  3  3 |

Start: Start position of the variant described in this record

End: End position of the variant described in this record

Tcn.em: Total copy number. For normal diploid Tcn.em is 2

**Table S4 Somatic CNV loss results**

| Chromosome | Start | End | Tcn.em |
| --- | --- | --- | --- |
| 1  3  3  3  4  4  4  4  4  5  5  5  8  8  8  8  9  10  10  13  13  13  15  15  15  17  18  18  18  18 | 245292527  2934700  60162400  177370500  20540652  37666300  41479300  44276000  181584300  26795  133385500  164998792  161900  2284111  8098577  12476200  141700  27366400  52934200  19036100  19657300  101287959  22681600  23650638  28434746  31212200  11358  38673987  63205217  72603843 | 249239500  3299866  60399400  197845600  37665300  41478238  41604500  49095200  190198703  34693  133874837  165147248  2190117  6824000  12014700  35962100  18069300  38806400  53322689  19652300  27004133  115056935  23642300  28425100  65546100  33699100  15162300  63204981  72602500  78010000 | 1  1  1  0  0  1  0  0  1  1  1  1  0  0  0  0  0  1  1  0  0  0  1  0  1  1  0  0  0  0 |

Start: Start position of the variant described in this record

End: End position of the variant described in this record

Tcn.em: Total copy number. For normal diploid Tcn.em is 2.

In all samples, an average of 3,325,047 SNPs were found, 98.37% of them appeared in the dbSNP database, and 93.98% were in the database of the 1000 Genomes Project. There were 53,785 SNPs newly discovered. The ratio of base conversion to base inversion is 2.02. In SNPs, compared to control cells, there were 53 synonymous mutations, 128 missense mutations, 10 nonsense mutations and 2 splicing mutations in the coding region. The statistical table of SNPs distribution for each sample was shown in Table S4.

**Table S5 Functional classification statistics of SNP in coding region**

| Samples | Synonymous | Missense | Stopgain | Stoploss | Startloss | Splicing |
| --- | --- | --- | --- | --- | --- | --- |
| 15μM PCB15  Control | 10063  10010 | 9826  9698 | 93  83 | 30  30 | 21  21 | 172  170 |

Synonymous: the number of mutations that cause the codon to encode the same amino acid; Missense: the number of mutations that cause the codon to encode different amino acids; Stopgain: the number of mutations that make a codon a terminating codon; Stoploss: the number of mutations that make a termination codon to a non-termination codon; Startloss: the number of mutations that make a start codon to a non-start codon; Splicing: the number of mutations at splicing sites.

On average, 993,240 Indels were found in all the samples; 72.38% of them appeared in the dbSNP database, and 42.80% in the database of the 1000 Genomes Project. There were 273,818 newly discovered Indels. In the newly discovered Indels, compared to control, there were 25 increased code-shift mutations in the coding region, and 11 Indels in the change of the splicing recipient or splicing donor of the splicing site region. The InDel distribution statistics for each sample and population was shown in Table S5.

**Table S6 Functional classification statistics of Indel in coding region**

| Samples | Frameshift | Non-frameshift Insertion | Non-frameshift Deletion | Stoploss | Startloss | Splicing |
| --- | --- | --- | --- | --- | --- | --- |
| 15μM PCB15  Control | 337  312 | 70  75 | 97  95 | 0  0 | 1  1 | 102  113 |

Frameshift: the number of insertions/deletions that cause code shift mutations in the coding region. Non-frameshift insertion: the number of insertions without causing a shift in the coding region. Non-frameshift deletion: the number of deletions without causing a shift in the coding region. Stoploss: the number of mutations that make a termination codon non-termination codon. Startloss: the number of mutations that make a start codon to a non-start codon; Splicing: the number of mutations at splicing sites.

**Table S7 Screening results of susceptible genes**

| Hugosymbol | CHROM | POS | REF | ALT | Variant-classification | AAchange | CGC-Cancers |
| --- | --- | --- | --- | --- | --- | --- | --- |
| PTCH1  BRCA2  BLM  ERCC4  BRCA1  BRCA1  SETBP1 | chr9  chr13  chr15  chr16  chr17  chr17  chr18 | 98209594  32914592  91326099  14029033  41223094  41244435  42456653 | G  C  C  G  T  T  G | A  T  T  A  C  C  A | Missense variant  Missense variant  Missense variant  Missense variant  Missense variant  Missense variant  Missense variant | PTCH1:NM_000264.3:p.Pro1315Leu/c.3944C>T  BRCA2:NM_000059.3:p.Arg2034Cys/c.6100C>T  BLM:NM_000057.3:p.Pro868Leu/c.2603C>T  ERCC4:NM_005236.2:p.Arg415Gln/c.1244G>A  BRCA1:NM_007300.3:p.Ser1634Gly/c.4900A>G  BRCA1:NM_007300.3:p.Glu1038Gly/c.3113A>G  SETBP1:NM_001130110.1:p.Ala222Thr/c.664G>A | Nevoid basal cell carcinoma syndrome  Hereditary breast/ovarian cancer  Bloom syndrome  Xeroderma pigmentosum  Hereditary breast/ovarian cancer  Hereditary breast/ovarian cancer  Schinzel-Giedion syndrome |

POS: the position on the chromosome; REF: refer to the base; ALT: alter to the base; AAchange: Amino acid change information; CGC-Cancers: cancer names in the CGC database.

The RNA-seq data showed that PCB153 exposure inhibited mismatch repair system (Figure S1), which might be associated with the genome-wide SNP and missense mutation events.


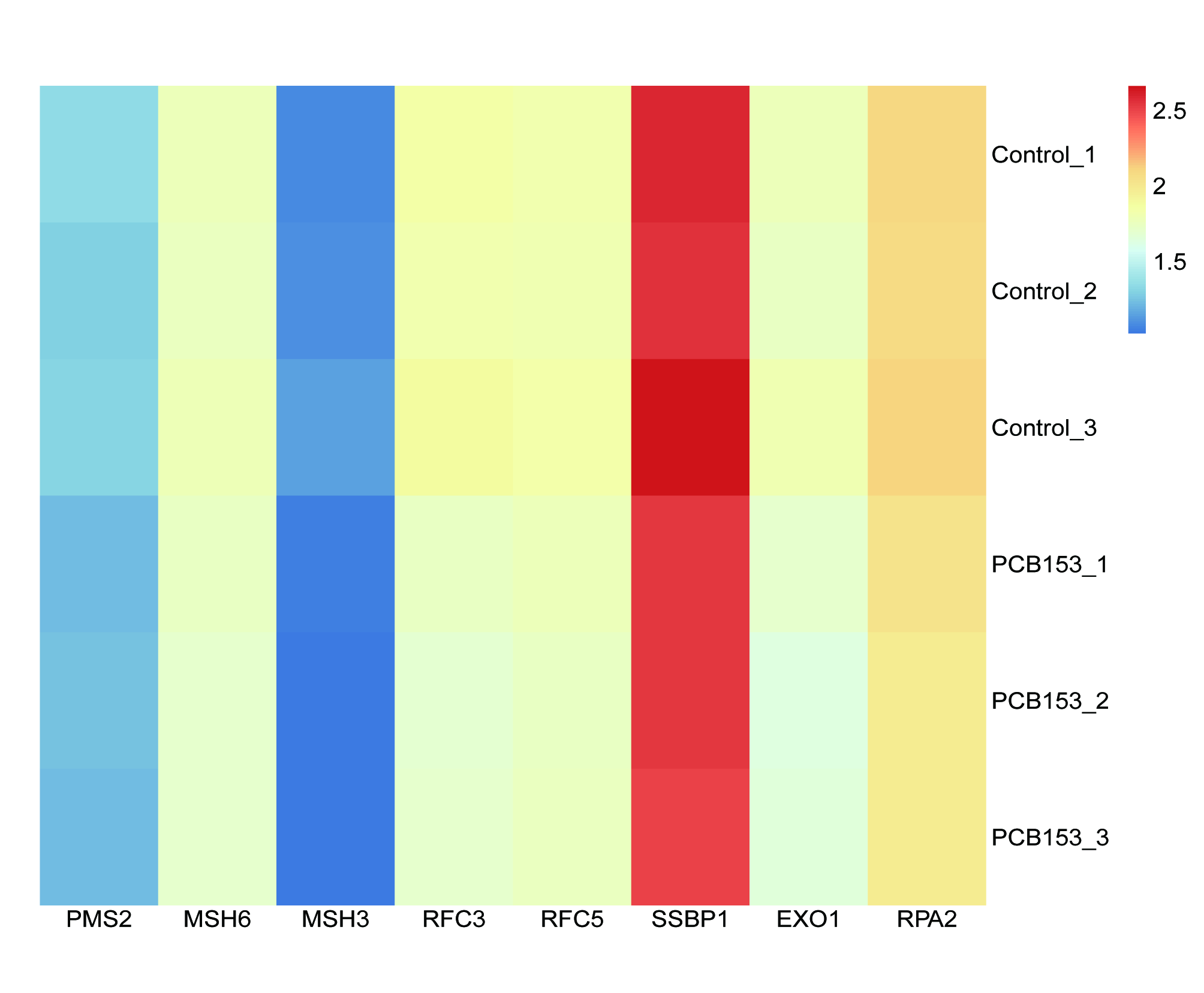


**Figure S1 The differentially expressed genes involved in mismatch repair of HEK293T cells post PCB153 treatment**

The heatmap showed the expression of genes involved in mismatch repair.

**
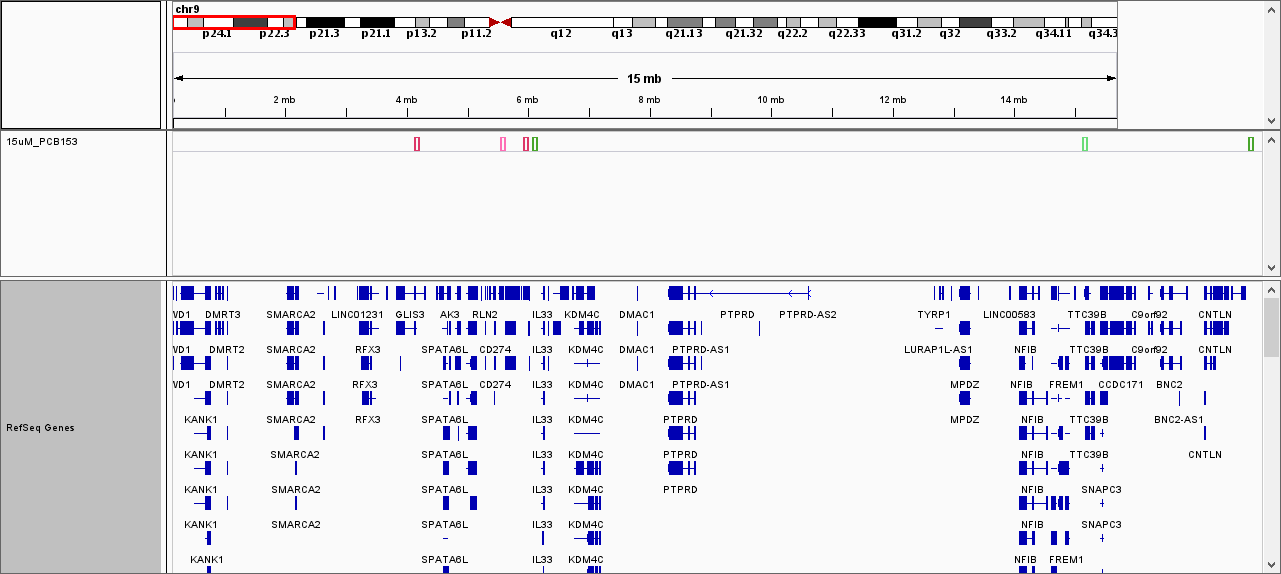
**

**Figure S2 Large fragment deletion region on chromosome 9**

The red frame showed the location of lost genes due to large fragment deletion on chromosome 9.

**
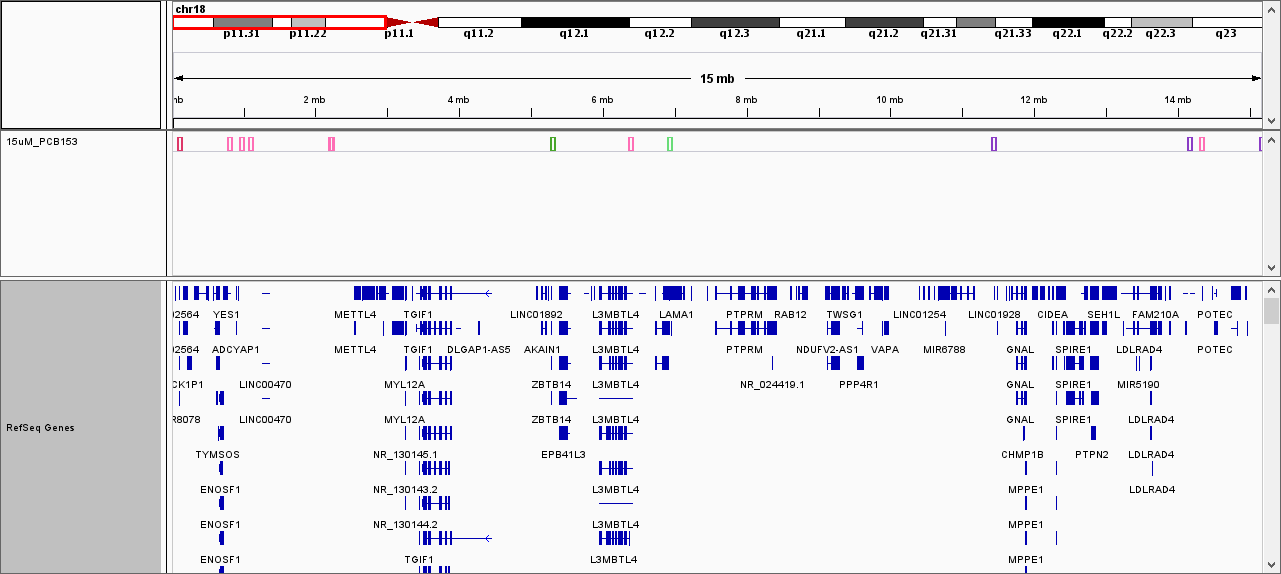
**

**Figure S3 Large fragment deletion region on chromosome 18**

The red frame showed the location of lost genes due to large fragment deletion on chromosome 18.


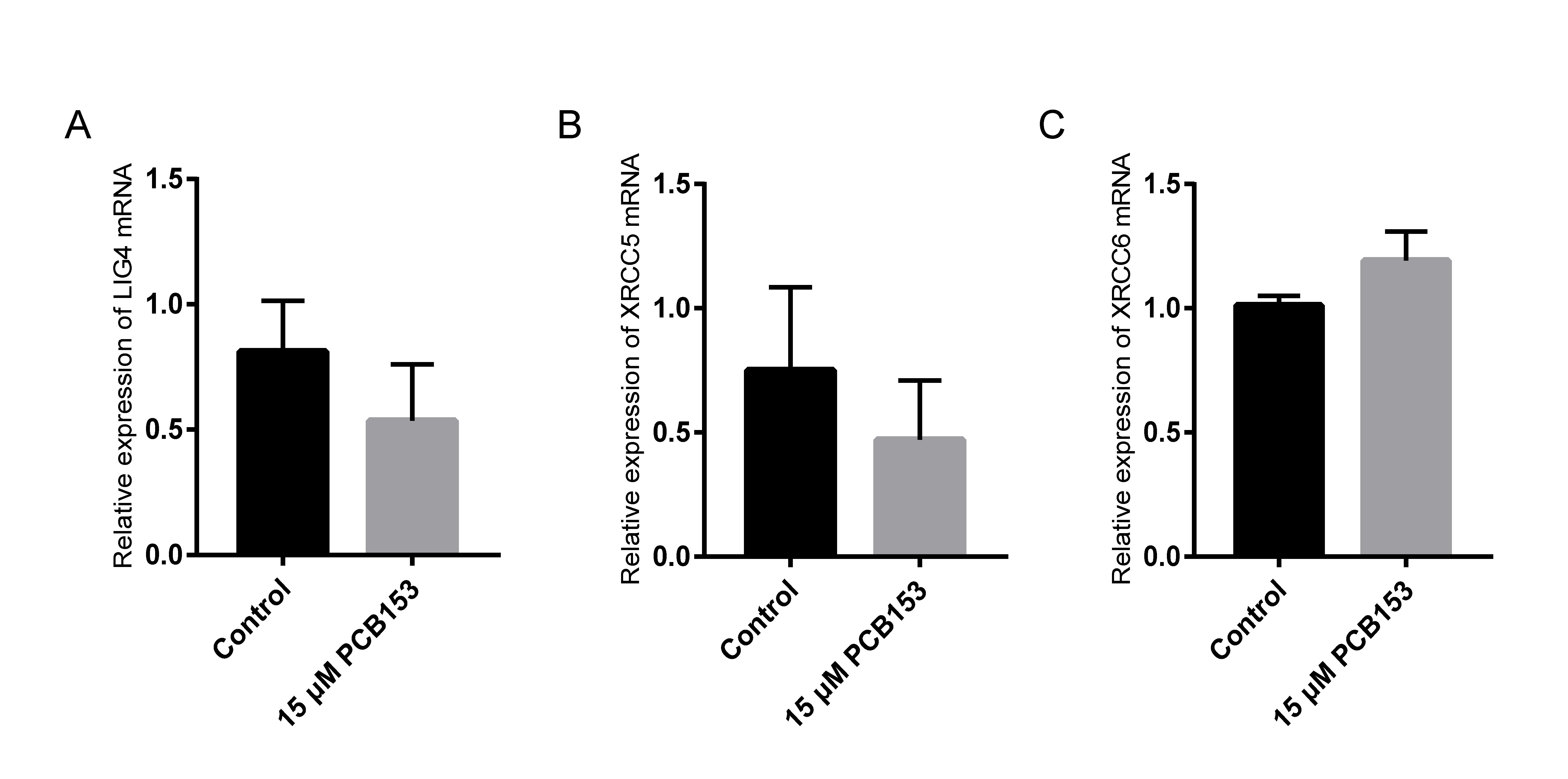


**Figure S4 NHEJ repair in PCB153-exposed HEK293T cells**

(A-C) Relative mRNA expressions of LIG4, XRCC5 and XRCC6 under 15 μM PCB153 treatment assessed using qPCR. The control cells were treated with 0.05% DMSO. Mean ± SD was shown (n = 3). Similar results were observed in at least three independent experiments.
